# Evaluation of an Adaptive Implementation Program for Cognitive Adaptation Training for People With Severe Mental Illness: a cluster-randomized controlled trial

**DOI:** 10.1192/j.eurpsy.2022.1948

**Published:** 2022-09-01

**Authors:** L. Van Der Meer, M. Van Dam, J. Van Weeghel, S. Castelein, G. Pijnenborg

**Affiliations:** 1 Lentis Psychiatric Institute, Department Of Rehabilitation, Zuidlaren, Netherlands; 2 University of Groningen, Clinical And Developmental Neuropsychology, Groningen, Netherlands; 3 Tilburg University, Tranzo Scientific Center For Care And Wellbeing, Tilburg, Netherlands; 4 Lentis Psychiatric Institute, Lentis Research, Groningen, Netherlands; 5 University of Groningen, Experimental Psychotherapy & Psychopathology, Groningen, Netherlands; 6 GGZ Drenthe, Department Of Psychotic Disorders, Assen, Netherlands

**Keywords:** psychosocial intervention, severe mental illness, cognitive rehabilitation, Implementation

## Abstract

**Introduction:**

Cognitive Adaptation Training (CAT) is a psychosocial intervention focusing on reducing the impact of cognitive disorders on daily functioning in people with severe mental illness (SMI). Similar to many evidence based practices (EBP), implementation of CAT in routine care lags behind, despite the established effectiveness of the intervention. This so called ‘science-to-service gap’ is a widespread problem in mental health care. We developed an innovative implementation program to facilitate implementation of CAT and similar interventions in routine care.

**Objectives:**

The aim of this study is to evaluate the effectiveness of the implementation program and to determine factors that impede or facilitate the implementation process.

**Methods:**

We conducted a multicenter cluster randomized controlled trial comparing the implementation program to a single training program in four mental health institutions (a total of 21 rehabilitation teams) in The Netherlands. Focus groups, semistructured interviews and questionnaires were used at multiple levels of service delivery (service user, professional, team, organization). Assessments took place before, during and after implementation and at follow-up, adding up to a total duration of 14 months. Data were analyzed using multilevel modeling.

**Results:**

Data collection is complete and analyses on the effectiveness of the implementation program are ongoing. Preliminary analyses show that team climate (p<.008) and organizational climate (p<.043) significantly predict the attitudes of mental health providers toward EBP.

**Conclusions:**

This implementation research may provide important information about the implementation of psychosocial interventions in practice and may result in a program that is useful for Cognitive Adaptation Training, and possibly for psychosocial interventions in general.

**Disclosure:**

No significant relationships.

